# Testis-Specific Isoform of Na^+^-K^+^ ATPase and Regulation of Bull Fertility

**DOI:** 10.3390/ijms23147936

**Published:** 2022-07-19

**Authors:** Saurabh Tiwari, Gayathri Rajamanickam, Veena Unnikrishnan, Mina Ojaghi, John P. Kastelic, Jacob C. Thundathil

**Affiliations:** Department of Production Animal Health, Faculty of Veterinary Medicine, University of Calgary, Calgary, AB T2N 4N1, Canada; saurabh.tiwari@ucalgary.ca (S.T.); gaya.rajamanickam@urus.org (G.R.); drveenaunnikrishnan@gmail.com (V.U.); mina.ojaghi@ucalgary.ca (M.O.); jpkastel@ucalgary.ca (J.P.K.)

**Keywords:** ATP1A4, capacitation, male fertility, signaling, sperm

## Abstract

An advanced understanding of sperm function is relevant for evidence-based male fertility prediction and addressing male infertility. A standard breeding soundness evaluation (BSE) merely identifies gross abnormalities in bulls, whereas selection based on single nucleotide polymorphisms and genomic estimated breeding values overlooks sub-microscopic differences in sperm. Molecular tools are important for validating genomic selection and advancing knowledge on the regulation of male fertility at an interdisciplinary level. Therefore, research in this field is now focused on developing a combination of in vitro sperm function tests and identifying biomarkers such as sperm proteins with critical roles in fertility. The Na^+^-K^+^ ATPase is a ubiquitous transmembrane protein and its α4 isoform (ATP1A4) is exclusively expressed in germ cells and sperm. Furthermore, ATP1A4 is essential for male fertility, as it interacts with signaling molecules in both raft and non-raft fractions of the sperm plasma membrane to regulate capacitation-associated signaling, hyperactivation, sperm-oocyte interactions, and activation. Interestingly, ATP1A4 activity and expression increase during capacitation, challenging the widely accepted dogma of sperm translational quiescence. This review discusses the literature on the role of ATP1A4 during capacitation and fertilization events and its prospective use in improving male fertility prediction.

## 1. Introduction

The sustainability of a burgeoning world population demands a concomitant rise in the efficiency of global food production [1]. Increased animal productivity substantially contributes to the World Health Organization’s sustainable development goals of zero hunger, good health, and wellbeing, which require the improved genetic selection of elite animals and widespread dissemination of their germplasm through reproductive technologies such as artificial insemination and embryo production. Furthermore, the success of these reproductive technologies is heavily dependent on fertility. Artificial insemination has substantially increased the rate of genetic gains using germplasm from one bull to breed numerous cows, making the fertility of an individual bull relatively more important than an individual cow [2].

Although a breeding soundness examination (BSE) can identify bulls that are grossly abnormal, this procedure is inadequate to identify sub-fertile bulls or predict variations in fertility among bulls that are considered fertile. A standard BSE investigates bull fertility based on conventional semen analysis, without considering submicroscopic differences in sperm characteristics affecting fertility [3]. The evaluation of one or more sperm functions in fertility prediction has been reported in various species. However, knowledge regarding the most suitable combination of parameters in fertility prediction is debatable [4], indicating the need to produce new knowledge on the molecular regulation of sperm functions. Bulls considered satisfactory based on a traditional BSE may differ in their fertility by 20–25% [5]. Therefore, it is desirable to use multi-parametric in vitro and in vivo tests to predict fertility, providing an impetus for research to decipher the molecular regulation of sperm function. 

In recent decades, semen evaluation has shifted towards an objective multi-parametric analysis using advanced techniques and multi-omic studies. This could assist in elucidating the reasons behind compromised semen quality in sub-fertile or infertile males, identifying biomarkers of fertility, and perhaps developing methods for early diagnosis of male subfertility/infertility. It could also be of interest in selecting young fertile bulls for commercial semen production and culling bulls with compromised fertility. Sub-fertile bulls cause considerable economic losses in terms of reduced conception rates, delayed calving-to-conception intervals, increased culling of females, etc. [2]. In several species, assisted reproductive techniques such as semen cryopreservation, artificial insemination, and in vitro embryo production have been extensively used for improving reproductive efficiency. However, technologies, where semen from a selected bull is extensively used for breeding, may propagate subfertility [6,7,8], and reduce genetic variability from the overuse of males with desirable traits [8].

During transit through the male and female reproductive tracts, dynamic microenvironments affect sperm function. For example, studies in rabbits [9,10], pigs [11], and cattle [12] demonstrated that temperature gradients in the female reproductive tract regulate sperm motility, capacitation, and fertilization. Similarly, sperm motility, viability, and glycolysis were modulated by pH and dissolved oxygen content in rabbits, rats [13,14], and humans [15]. During this transit, sperm functions are regulated by the dynamic regulation of ions along the sperm membrane. In the epididymis, mammalian sperm are quiescent and undergo maturational changes, where sperm-specific ion channels have an important role in attaining sperm motility during ejaculation (reviewed by [16]). Ion channels regulate sperm membrane potential, cytoplasmic Ca^2+^ concentration, and intracellular pH, aiding molecular events such as capacitation, acrosome reaction, and hypermotility [17,18]. Several sperm-specific ion channels such as CatSper, Na^+^-K^+^-ATPase (NKA), Ca^2+^-activated Cl^−^ channels, and voltage-gated H^+^ channels have been studied for their roles in sperm physiology. The role of NKA in attaining sperm-fertilizing potential is crucial and well documented in cattle [3,19,20], rats/mice [21,22,23], and humans [24,25]. During the capacitation of hamster sperm, hyperactivated motility is chiefly regulated by extracellular Na^+^ concentrations [26]. In addition to NKA, the Na^+^/Ca^2+^ (NCX) ion channel also participates in Na^+^ homeostasis; however, NCX (voltage-dependent channels) functionality is reliant on the electrochemical gradient created by NKA [21]. This review discusses the literature on sperm-specific NKA α4 isoform (ATP1A4) and its role in signal transduction, the regulation of sperm functions, and male fertility across species. 

## 2. Na^+^-K^+^ ATPase (NKA) Ion Channel

A ubiquitous heterodimeric transmembrane protein first described by Jens Christian Skou [27], NKA has two amphipathic α and β subunits. The α subunit contains ~1012 amino acids (110 kDa) with almost identical sequences across species and tissues [28]. In vertebrates, three isoforms of α subunit have been identified: isoform α1 is ubiquitous in all mammalian tissues, α2 predominates in skeletal muscle, α3 is in the brain and nervous tissues together with α1 and α2 [28], and α4 (ATP1A4) is only in male germ cells [29,30,31,32]. The other β subunit contains ~300 amino acids (35 kDa) and has three isoforms (β1, β2, and β3), with a low homology in amino acid sequences across species and tissues [33]. 

The NKA exists in cell membranes as an (αβ)_2_ diprotomer; the two subunits are difficult to separate without the loss of enzymatic activity [34]. The α subunit governs the ATP hydrolytic activity of NKA for Na^+^ and K^+^ transport and comprises the ouabain binding site that specifically inhibits the enzymatic function and stimulates the signaling task of NKA [35]. In addition, the β subunit provides a structural role in the dimeric form and regulates the number of sodium pumps transported to the plasma membrane through α and β heterodimer assembly [36]. 

## 3. NKA in Somatic Cells

NKA exists in the plasma membrane in two functionally distinct pools, with one involved in Na^+^ and K^+^ transport across the plasma membrane (pumping pool) and the other involved in cell signaling (non-pumping pool) [37]. NKA (pumping pool) helps to maintain the resting membrane potential and action potential through ionic gradients across the plasma membrane, with three Na^+^ exchanged from inside to the outside and two K^+^ from outside to inside the cell. These gradients also facilitate cell homeostasis, such as regulating cell volume and cytoplasmic pH through Na^+^/H^+^ antiport, Cl^−^/HCO_3_^−^ exchange, and Na^+^-HCO_3_^−^ co-transport, while regulating intracellular Ca^2+^ concentrations through the Na^+^/Ca^2+^ antiport [38]. 

The NKA α subunit has a ouabain-binding site in the extracellular side of the transmembrane cleft where ouabain binds to both pumping and non-pumping pools [37]; however, the physiological manifestation of its binding is dose-dependent. Ouabain inhibits the actions of the pumping pool at higher (millimolar) concentrations, whereas the non-pumping pool is inhibited by lower (nanomolar) ouabain concentrations [39]. The ouabain concentration necessary to activate the signaling pathways varies with species, as evidenced by a lower ouabain concentration (two to three times) eliciting equivalent effects in human cell lines compared to rodent cell lines [40,41,42]. Moreover, ouabain binds to various isoforms of NKA α subunits with differential affinity, i.e., α1 being 100-fold more resistant to ouabain binding than α2 and α3 isoforms in rats [43,44,45]. The dimeric state of various isoforms of α and β subunits affected ouabain binding in a murine fibroblast cell line, with α3β1 and α3β2 having a high sensitivity to ouabain, α2β1 and α2β2 intermediate, and α1β1 low [46]. 

The ouabain inhibition of NKA pump increases [Ca^2+^]_i_ without affecting signal transduction, indicating the NKA non-pumping pool function is independent of intracellular Na^+^ and Ca^2+^ ion concentrations [47]. The NKA non-pumping pool apparently resides in cholesterol-rich membrane microdomains, i.e., lipid rafts and caveolae [48], where it directly interacts with protein kinases, ion transporters, and structural proteins to exert its non-pumping functions. Lipid rafts could be planar/non-caveolar rafts with non-invaginated microdomains or caveolae with tube-like invaginations in the plasma membrane, acting as a platform for protein endocytosis and trafficking [49]. Ouabain binding to the non-pumping NKA pool induces protein and lipid kinase cascades and generates several secondary messengers [50,51,52,53]. Ouabain interacts with the NKA α subunit to activate the EGFR/Src-Ras-ERK [40,50,53] or PI3K1A-PDK-Akt pathway [54,55], thereby stimulating tyrosine phosphorylation of downstream effectors, activating protein kinase cascades and generating secondary messengers. NKA α1 isoform knockdown reduced the size of a pool of Src-interacting Na/K-ATPase, implying loss of the “non-pumping” pool involved in cell signaling while preserving the pumping pool [37]. With the disruption of lipid rafts and caveolae, interacting proteins (or factors) are removed and a portion of non-pumping NKA is converted to a pumping fraction [37]. 

## 4. Distribution of ATP1A4 in the Testis and Sperm 

Various NKA isoforms of α subunit have been identified in the epithelium of the seminiferous and epididymal tubules, and germ cells in the male reproductive tract. Testes in rats, humans, and cattle contain exclusively α1 and α4 isoforms [19,29,56], whereas sperm differ in the presence of α1, α2, α3, and α4 isoforms in a species-dependent manner. During rat germ cell differentiation, α1 expression displays only a modest change; however, its relative contribution to total NKA activity is significantly decreased [22]. In contrast, ATP1A4 expression and activity are significantly increased throughout spermatogenesis. The ATP1A4 mRNA levels peaked in pachytene spermatocytes and round spermatids, whereas protein levels peaked in rat sperm [22], indicating distinct regulation of each NKA isoform during gametogenesis.

ATP1A4 localization on the sperm plasma membrane is species-specific, primarily in the flagellum in most species with distinct head compartmentalization in bovine sperm. In rat and human sperm, ATP1A4 is mainly localized in the mid-piece and principal piece of the flagellum, respectively, with no or little α4 isoform in the head [29,30,57]. In bulls, ATP1A4 is mainly expressed in the sperm head; however, it localizes differentially with capacitating conditions [20]. This protein is re-localized from the acrosomal region in fresh (uncapacitated) sperm to the equatorial segment and post-acrosome region during capacitation. The other subunits, α1 and α3, are present primarily in the equatorial region and post-equatorial regions, respectively, of bovine sperm [58]. In contrast, α2 and α3 isoforms are not expressed in rat, mouse, or human sperm [58]. 

## 5. Role of NKA α4 Isoform in Sperm Physiology

The discovery of proteins specific to the testis and sperm has advanced understanding of sperm functions and regulation of male fertility. A sperm-specific protein, ATP1A4, has a crucial role in the regulation of mouse sperm motility [19,52], capacitation [59] and oocyte binding and activation by Phospholipase C zeta (PLC ζ) in bull sperm [60]. ATP1A4 is less influenced by changes in extracellular Na^+^ and temperature than the α1 subunit; perhaps ATP1A4 can regulate ionic gradients during capacitation without being strongly inhibited by hyperpolarization and extracellular sodium [61]. ATP1A4 is essential for fertility, as evidenced by the complete sterility in knockout mice [21]. Moreover, ATP1A4 activity exceeded α1 isoform by at least twofold in rats [22]. The in vivo fertility of high-fertility (HF) and low-fertility (LF) bulls has been associated with ATP1A4 content and enzymatic activity, which were higher in HF versus LF bulls [62]. Immunoblots of ouabain-induced capacitated sperm from HF bulls had a higher band intensity of tyrosine phosphorylation than LF bull sperm, suggesting a differential predisposition in the capacitation-associated signaling mechanism [62]. It was presumed that either sperm from LF bulls have a lower inherent ATP1A4 content, or they incur higher plasma membrane damage during freeze–thawing, thereby experiencing higher ATP1A4 loss than sperm from HF bulls [3]. 

### 5.1. ATP1A4-Associated Signaling Pathways Involved in Bull Sperm Capacitation

Capacitation is a maturation process undergone by ejaculated sperm in the female reproductive tract for a species-dependent interval to achieve fertilizing ability [63]. Multiple physiological and biochemical changes occur in sperm during capacitation viz. increased membrane fluidity, lateral cholesterol migration to the apical area of the sperm head, and cholesterol efflux from the plasma membrane [64], remodeling of actin, hyperactivated motility [65], etc. During capacitation, sperm have a high amplitude, asymmetrical flagellar beating pattern called hyperactivation [63].

Several biomolecules in secretions of the female reproductive tract viz. albumin [66], heparin, ouabain [67], sterol sulphatase [68], progesterone [69], and uterine and oviduct proteins (reviewed by [70]) modulate sperm physiology to acquire fertilizing capacity. Characteristics of NKA channel inhibition by ouabain, a cardiac glycoside, have been explored across species to understand the role of ATP1A4 in sperm functions. However, this section focuses on ouabain-induced ATP1A4 signaling in bovine sperm. The presence of ouabain in bovine vaginal fluid in nanomolar concentrations [67] indicates its association with sperm NKA, thereby modulating sperm physiology in the female reproductive tract during biochemical events such as capacitation.

Ouabain binds to various isoforms of NKA α subunits with differential affinity [39]. The NKA subunit contains two ouabain binding sites: a low-affinity binding site between transmembrane (TM) regions TM 1 and TM 2, and a high-affinity binding site between TM 4 and TM 6, which differ only by a few amino acids [71]. A recent study modified the ouabain affinity of ATP1A4 and NKA α1 in mice and detected no effect on the reproductive phenotype, concluding the high-affinity ouabain binding sites of NKA to be insignificant for mouse sperm fertility [23]. This might be due to the lack of a direct relationship between high-affinity sites and ouabain-induced signaling in mouse sperm; however [23], the same should also be investigated in livestock species.

The testis-specific NKA α4 isoform has a higher sensitivity to ouabain than other isoforms [30], e.g., the ouabain affinity of α4 isoform is approximately 1000-fold higher than that of α1 isoform in rat sperm [22]. This differential sensitivity to ouabain has been used for a dose-dependent ATP1A4 inhibition in understanding its specific role in sperm functions. ATP1A4 is the predominant catalytic subunit of NKA, which accounts for two-thirds of the total sperm NKA activity [22]. However, the α2 isoform has recently been reported to be the predominant isoform on the raft fractions in bovine sperm head plasma membrane during ouabain-induced capacitation [72]. 

**Figure 1:**
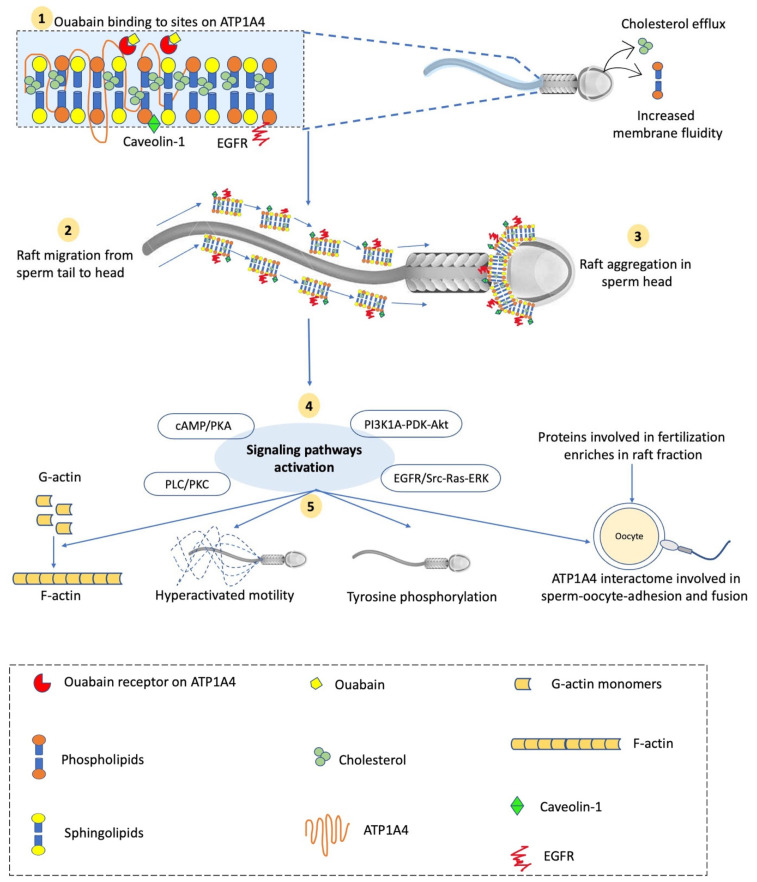
Schematic diagram of events during ouabain-induced signaling during bovine sperm capacitation. (**1**) Ouabain binding to its sites on ATP1A4 induces capacitation, where ATP1A4 interacts with caveolin-1 and EGFR in the raft fraction. (**2**) Simultaneous sperm surface alterations such as cholesterol efflux and increased membrane fluidity result in sequential raft migration from sperm tail to head. (**3**) Consequently, there is raft aggregation in the post-acrosome and equatorial segment of the sperm head, enriching proteins involved in fertilization events and providing a platform for signaling molecules to activate downstream effects. (**4**) Various signaling pathways are activated and polymerize G-actin to F-actin with concomitant tyrosine phosphorylation of proteins and sperm hyperactivation. (**5**) ATP1A4 interactome on sperm surface interacts with zona pellucida to facilitate sperm–oocyte adhesion and fusion.

In bull sperm, ouabain interacts with NKA to induce the tyrosine phosphorylation of intracellular proteins and capacitation [20,59]. ATP1A4 activates the specific downstream signaling molecules caveolin-1 and EGFR in the raft fraction (Figure 1) and Src, EGFR, and ERK1/2 in the non-raft fraction of the sperm plasma membrane under ouabain-induced capacitating conditions [73]. As a result, during mammalian sperm capacitation, signaling pathways viz. the cAMP/PKA pathway, PLC/PKC pathway [74,75], PI3K/Akt pathway [76], and ERK 1/2 pathway [20,77] are activated. Ouabain interacts with NKA and induces protein tyrosine phosphorylation by activating the ERK1/2 (potentially ERK2) signaling pathways, which essentially require ATP binding to Src. In contrast, heparin induces capacitation and activation of the ERK1/2 signaling pathway primarily through the cAMP/PKA pathway in an Src-independent manner [78]. Moreover, ouabain competes with progesterone to bind low-affinity ouabain binding sites on the NKA α1 subunit in bull sperm [79] and amphibian oocytes [80]. Ouabain induces capacitation and tyrosine phosphorylation more effectively than progesterone; however, it has a lower binding affinity (in vitro) to these sites than progesterone [79]. Moreover, the capacitation-associated changes were higher in HF versus LF bulls. This variation may be attributed to the stimulation of various signaling pathways involved in capacitation. Like ouabain, progesterone also stimulates Ca^2+^-induced PLC-DAG/IP3-PKC and MAPK pathways; however, the cAMP/PKA pathway is not activated [81]. It also indicates that the amount/distribution of various NKA isoforms (α1, α2, α3) may vary with bull fertility and warrants further investigation [82]. Contrary to ouabain, the use of another NKA inhibitor, digoxin, was observed to have a temporal effect in inducing bovine sperm capacitation, where 2 h of incubation with digoxin concomitantly reduced the sperm protein tyrosine phosphorylation state and percentage of full-type hyperactivated sperm [83]. The addition of cAMP analog cBiMPS and protein phosphatase inhibitor calyculin A reduced this temporal effect and significantly increased the percentages of full-type hyperactivation for semen samples with low survivability [83]. However, the effects of digoxin on intracellular Ca^2+^-dependent signaling cascades during capacitation need further investigation.

### 5.2. ATP1A4 Interactome in Sperm Raft and Non-Raft Fractions during Capacitation

Cholesterol is an integral component of the plasma membrane, which significantly affects its physical properties. Cholesterol orders the lipid bilayer in one dimension and reduces its permeability; however, the lateral diffusion rate of lipids and proteins in the plane of the bilayer is minimally affected [84]. The plasma membrane in somatic cells contains lipid rafts, which are domains within lipid bilayer enriched in cholesterol, sphingomyelin, glycosphingolipids, and saturated phospholipids [85]. The lipid raft mediates signal transduction between proteins from the exoplasmic leaflet to the inner leaflet on the plasma membrane, resulting in a cellular response [86]. Moreover, it is argued that lipid rafts allow activated receptors enhanced access to specific downstream signaling proteins involved in signal transduction, and interactions with unrelated proteins [87].

The presence of raft and non-raft fraction in sperm plasma membrane has been widely reported in sperm from mice [88], pigs [89], bulls [20], chickens [90], and humans [91]. Although cholesterol helps in lipid raft stabilization, regulated low-level cholesterol efflux from the sperm plasma membrane during capacitation does not affect the raft composition [92,93]. However, a polarized migration of lipid rafts takes place sequentially from sperm tail to head during capacitation in boars [89], with the concomitant phosphorylation of intracellular proteins in bull, boar, and ram sperm [94]. Interestingly, this polarized lipid raft migration did not cross the boundary between the post-acrosome and equatorial segment in the sperm head, indicating the presence of a molecular filter allowing the free movement of single molecules, but not larger complexes such as lipid rafts [94]. The lipid raft migration is preferentially stimulated by the regulated loss of cholesterol from the non-raft pool, which promotes the coalescence of microdomains into large micrometer-scale domains [95]. This is crucial to place lipid rafts in the appropriate position in the sperm head to activate the downstream signaling pathways involved in the capacitation and exocytosis of acrosome vesicles. Similarly, a cholesterol loss-dependent shift of GM1 and CD59 proteins (lipid raft markers) from the raft to the non-raft fraction was reported during the capacitation of human sperm [91]. These changes activate the signal transduction pathway involving protein kinase A and tyrosine kinase second messenger systems, subsequently resulting in protein tyrosine phosphorylation [96]. Moreover, raft reordering in the boar sperm surface generates the protein complexes involved in zona pellucida binding [92,97]. When excessive cholesterol is removed from the sperm plasma membrane, it disrupts the lipid rafts and decreases tyrosine phosphorylation [94].

In addition to signaling molecules, lipid rafts in sperm contain several proteins that regulate sperm functions and fertilization events. Previous studies reported a differential protein enrichment in raft and non-raft fractions of sperm membranes that mediate sperm-oocyte interactions in vertebrates [98,99] and invertebrates [100], such as acrosin, PH-20, basigin, and the cysteine-rich secretory protein 1 [101,102,103]. The presence of egg–zona binding proteins in the sperm raft fraction such as CD59, fertilin-β, AQN-3/spermadhesin, and P47/SED-1 suggests that they have major roles in fertilization events [92,97]. Other proteins with zona-binding affinity such as arylsulphatase A [104], testis-specific isozyme of angiotensin-converting enzyme (tACE) [105], acrosomal vesicle protein 1 [106], zonadhesin [107], and Zona Pellucida Binding Protein-1 (ZPBP1) [108] also aggregate in raft fractions in the head of capacitated sperm. Moreover, lipid rafts enrich a variety of ion transporters/channels. For instance, plasma membrane Ca^2+^ ATPase is enriched in sperm lipid raft fractions from chickens [90], bulls [109], mice [98], and humans [99], which significantly contributes to the induction of an acrosome reaction and hyperactivated motility [110]. 

Another vital ion transporter in the sperm plasma membrane is NKA, which mainly resides in lipid rafts, facilitating cell signaling due to its proximity to other signaling molecules within these microdomains/rafts [30]. Ushiyama et al. [90] reported that lipid rafts in the chicken sperm membrane were enriched in NKA isoforms (α1, α3, β1). Various NKA isoforms involved in capacitation-associated signaling have been demonstrated in both raft and non-raft fractions of the plasma membrane of bull sperm. All of the NKA isoforms are present in the raft and non-raft fractions of head plasma membrane in bull sperm, and among various isoforms (α1, α2, α3, β1, β2, and β3) in the raft fraction, α3 and β1 were the most abundant isoforms [72]. The existence of α4 isoform has also been demonstrated in sperm raft and non-raft fractions, where its content increases during capacitation [20]. During ouabain-induced capacitation in bovines, ATP1A4 interacted with caveolin-1 and EGFR in the raft fraction, and Src, EGFR, and ERK1/2 in the non-raft fraction; however, ATP1A1 only interacted with caveolin-1 in both fractions of capacitated and uncapacitated sperm [73]. Several proteins viz. hexokinase 1, plakophilin 1, desmoglein 1, 14-3-3 protein/, cathepsin D, and heat-shock protein 1 (HSP1) were specific to the non-raft component of the ATP1A4 interactome, whereas glutathione S-transferase and annexin A2 were exclusive to the raft interactome. However, ADAM32, histone H4, actin, acrosin, serum albumin, and plakoglobin were common to both raft and non-raft fractions [73]. These proteins in the ATP1A4 interactome are involved in various biological processes, e.g., fertilization, signal transduction, cell–cell adhesion, metabolism, and motility, and their roles in sperm–oocyte adhesion and fusion are discussed in subsequent sections. Interestingly, ATP1A4 translocated from the anterior acrosome to the equatorial segment and post-acrosomal regions following capacitation, and merged with the plakoglobin signal in the equatorial segment, implying interactions during downstream capacitation events [73]. 

### 5.3. ATP1A4 Function in Sperm Motility and Capacitation-Associated Hyperactivation 

The NKA generates an electrochemical potential gradient across the plasma membrane that is utilized by NCX channels [111] and Na^+^/HCO_3_^−^ cotransporter [112] to increase intracellular Ca^2+^ and HCO_3_^−^, respectively. The Ca^2+^ and HCO_3_^−^ coordinate the stimulation of soluble adenylyl cyclase and subsequent synthesis of cAMP, the activation of protein kinase A, phosphorylation of tyrosine residues, and hyperactivation. Ouabain-induced ATP1A4 inhibition (at a dose of 10^−6^ M) inhibited hyperactivation without affecting the percentage of motile sperm in hamsters [113], implying ATP1A4 primarily regulates capacitation-associated hypermotility [57]. ATP1A4 increases flagellar bending and decreases flagellar beat frequency during hypermotility; however, it does not affect the total sliding of microtubules in hamster sperm [113]. Conversely, the Na/K-ATPase α1 isoform maintains basal motility, i.e., an impulse produced by the transverse waves along the flagellum in a proximal–distal direction, assisting sperm to traverse the female genital tract [113,114]. However, an indirect role of ATP1A4 in regulating basal motility has also been reported. ATP1A4 indirectly regulates a rise in intracellular H^+^ during active sperm movement via the Na^+^/H^+^ exchanger (NHE) [115]. The sperm flagellar bending pattern and its response to cAMP and Ca^2+^ are modulated by intracellular [H^+^] [116]. Ouabain-induced selective ATP1A4 pump inhibition decreased intracellular pH and eliminated rat sperm motility [21], which was regained by inducing H^+^ movement out of cells with the ionophores nigericin and monensin [30]. Moreover, the co-localization of NHE1 and NHE5 with ATP1A4 supports its role in maintaining rat sperm motility [30].

The maintenance of [Ca^2+^]_i_ in a limited range is also vital for sperm motility [117] and is indirectly regulated by NKA through NCX channels [21]. The curvature and symmetry of the sperm flagellum are affected by changes in free (intracellular) calcium [116], thereby affecting motility. The N-terminal of the NKA α subunit directly interacts with IP3 receptors, indicating ouabain-induced conformational changes can directly increase intracellular Ca^2+^ concentrations [118,119]. 

The differential sensitivity of various NKA isoforms to ouabain and its dose-dependent effect on sperm functions have been widely explored to understand ATP1A4 role in sperm motility. Ouabain inhibits the NKA enzymatic activity at higher concentrations (milli-molar) but stimulates signaling pathways at lower (nanomolar) concentrations [39]. The ouabain-mediated ATP1A4 inhibition increased [Ca^2+^]_i_ through a reduced cation clearance, and decreased sperm kinematics in rats in a time-dependent manner [21]. Interestingly, NCX is expressed concurrently with ATP1A4 in the mid-piece of rat sperm [30,57,117]. The NCX and ATP1A4 activity were reported to be lower in asthenozoospermic infertile couples than normozoospermic couples. In the latter, ATP1A4 was localized in both sperm head and tail; however, in asthenozoospermic couples, its localization was only detected in the sperm head and was absent in the tail, indicating that ATP1A4 is associated with a sperm motility disorder [120]. In contrast, the ouabain-induced inhibition of NKA activity decreased progressive motility without any effect on [Ca^2+^]_i_ in bull sperm [59]. Moreover, hyperactivation [113], tyrosine phosphorylation, and capacitation are not affected by ouabain-induced NKA inhibition [59]. This variation may be due to the calcium-independent activation of MAPK signaling and tyrosine phosphorylation similar to somatic cells [54], differences in ouabain concentrations, or incubation time among experiments or species-specific differences. 

### 5.4. AT1A4 in Sperm–Oocyte Interaction and Activation

During capacitation, ATP1A4 interacts with several sperm proteins in raft and non-raft fractions of the plasma membrane (e.g., hexokinase, actin, and plakoglobin), which are assumed to facilitate sperm–oocyte interaction and activation. In this direction, Rajamanickam et al. [73] proposed a model explaining possible molecular interactions during sperm–oocyte interaction. Ouabain-induced sperm capacitation activates the EGFR signaling pathway, followed by Src activation, which in turn results in tyrosine phosphorylation, co-localization of PLC ζ and ATP1A4 to the post-acrosomal region of the sperm head, and PLC ζ activation [3,60]. Concurrently, ATP1A4 binds to ankyrin (an anchor protein), which mediates its interaction with the actin cytoskeleton, thereby facilitating contact with F-actin–plakoglobin–E-cadherin complex on sperm membrane [73]. Thereafter, the complementary E-cadherin molecules on sperm in the equatorial region of the sperm head and the microvillar region on oolemma [121] would bind and augment sperm–oocyte interaction [3]. This would promote PLC ζ entry from the perinuclear theca region of the sperm [122,123] to the oocyte, which would catalyze the hydrolysis of PIP_2_ to DAG and IP_3_, thereby releasing intracellular calcium from the endoplasmic reticulum, leading to calcium oscillations [124]. Consequently, metaphase II-arrested oocyte resumes meiosis, the second polar body is extruded, and a female pronucleus is formed [125]. The sperm nucleus decondense and result in male pronucleus formation. The fusion of male and female pronuclei results in zygote formation. Lestari et al. [25] reported low sperm NKA activity to significantly affect embryo development and cleavage (two-cell and eight-cell stages) following intracytoplasmic sperm injection (ICSI) and suggested the use of NKA activity in screening sperm for ICSI. Moreover, the under-expression of ATP1A4 and other proteins in testicular cancer seminoma was associated with a decreased fertilizing ability of affected men [126]. The proposed mechanism of sperm–oocyte interaction may assist in investigating pathological and unexplained male subfertility/infertility.

## 6. De Novo ATP1A4 Translation during Capacitation

Sperm are considered transcriptionally and translationally inactive; however, several studies have provided insights into de novo protein synthesis. Capacitation involves a complex set of highly regulated molecular and physiological events and is an extensively studied phenomenon in sperm biology. Sperm may require a new set of proteins or more of the existing proteins for capacitation, indicating protein synthesis in sperm from existing transcripts. During capacitation, the redistribution of Angiotensin II and progesterone receptors to various sperm regions and its association with changes in total sperm protein was demonstrated in humans [127]. Another observation of an increase in the total content of ATP1A4 in both raft and non-raft fractions of the sperm plasma membrane provided evidence of de novo protein synthesis in mature bull sperm during capacitation [20]. 

The incorporation of the fluorescent amino acid (lysine transfer RNA labeled with fluorophore BODIPY-FL and [35S] Met–[35S] Cys) during capacitation into nascent proteins was clear evidence that sperm are translationally active [20,128]. Recently, the incubation of sperm in capacitating medium changed the relative abundance of the sperm proteins involved in motility, fertilization, energy production, and signaling [129]. Moreover, the induction of an acrosome reaction also reduced the abundance of proteins involved in sperm–oocyte recognition, binding, and fusion [129]. There was a debate as to whether the change in the relative abundance of proteins was due to dynamic molecular changes such as protein modification, degradation, or translocation, and did not involve sperm translational activity. However, the argument was not validated by investigating protein synthesis in sperm. Another study reported boar sperm capacitation to induce differential expression of microRNAs and mRNAs than uncapacitated sperm [130], suggesting that sperm may require a new set of proteins for this physiological event.

In contrast to somatic cells, mature sperm are devoid of cytoplasm [131], and therefore, the existence of translational machinery (i.e., ribosomes) is questionable. However, the evidence of sperm protein synthesis indicates a gap in understanding the mechanisms underlying sperm translation. In preliminary studies, translation in mature bovine sperm was mitochondrial in origin and did not require transcription and translation of nuclear information [132]. Furthermore, this indicated the importance of existing sperm transcripts and challenged the view that mature sperm have all of the proteins required for successful fertilization. 

## 7. ATP1A4 as a Potential Candidate Biomarker in Male Fertility Prediction 

Male fertility is regulated by several factors and, therefore, a BSE or conventional semen analysis is inefficient in predicting fertility variations among bulls. It is also noteworthy that a bull’s ejaculate does not represent a uniform, homogeneous sperm population; rather, it consists of subpopulations with different functional characteristics such as motility [133,134,135], morphology [136,137], energetics (substrate use, mitochondrial activity, or ATP content) [138], protein, and RNA content [139,140], etc. Interestingly, single-cell sequencing revealed that each spermatozoon in an ejaculate has a unique genome, which accounts for its exclusive functional characteristics in an ejaculate [141]. Therefore, an improved understanding of physiological events such as capacitation and sperm–oocyte interaction could reveal potential biomarkers that could not only predict male fertility, but also the fertilizing potential of a spermatozoon, thereby improving the efficiency of ARTs such as ICSI. Single-nucleotide polymorphism markers, differential protein and RNA expression, and metabolite composition have been investigated in high- and low-fertility bulls using a multi-omics approach; however, we have only discussed here the literature regarding protein biomarkers. 

Male fertility prediction is investigated either as a negative or positive biomarker-based approach. A negative biomarker-based approach is based on identifying proteins or ligands unique to defective spermatozoa and aims to identify and remove infertile and sub-fertile bulls from the breeding herd, whereas the positive biomarker-based approach involves the selection of HF breeding bulls. Proteins such as TMEM95 [142], Postacrosomal Sheath WWI Domain Binding Protein (PAWP) [143,144,145,146], ubiquitin [144], and ubiquitinated arylsulfatase A [104] are associated with poor fertility and can be used to monitor breeding programs with low pregnancy rates. In contrast, proteins like phosphatidylethanolamine-binding protein 4 (PEPB4), which is absent in infertile bulls [147], or testis-specific isozyme of angiotensin-converting enzyme (tACE), whose activity and content was higher in HF bulls than LF bulls [105], can serve as candidate biomarkers for a positive biomarker-based approach. Several factors strengthen ATP1A4 as a potential candidate for male fertility prediction, such as its germ cell-specific nature of ATP1A4, essential role in sperm capacitation, oocyte binding, and interaction with other proteins involved in fertilization events such as t-ACE [148] and PLC ζ [60] higher ATP1A4 activity and content in HF than LF bulls [62]. Since fertility regulation is multifactorial, a combination of biomarkers can increase the accuracy of male fertility prediction.

## 8. Conclusions and Future Directions

The prediction of male fertility requires a multifactorial approach, with an assessment of submicroscopic differences in sperm essential to improve our precision. The significance of ATP1A4 in sperm physiology associates well with the current focus on identifying sperm proteins as biomarkers for improved fertility prediction and addressing male infertility. During capacitation, lipid raft aggregation enriches the proteins involved in fertilization events where ATP1A4 interacts with signaling molecules to regulate capacitation-associated events such as sperm motility, tyrosine phosphorylation, and hypermotility, and potentially contributes to the PLC ζ-mediated activation of oocytes. ATP1A4 re-localization in the lipid rafts of the sperm head is essential for interacting raft/non-raft fraction proteins to activate signaling pathways; however, further investigation of the specific mechanisms of action of ATP1A4 in the male gamete physiology leading to fertilization is needed. Moreover, an increase in ATP1A4 protein during capacitation challenges the widely accepted dogma of sperm translational quiescence. The exclusion of translational machinery (ribosomes) with cytoplasm at the end of spermatogenesis demands investigating the underlying mechanisms regulating the translation of this protein during capacitation. An improved understanding of proteins regulating sperm functions at the molecular level may assist in differentiating apparently normal sperm through conventional semen evaluation methods. 

## Data Availability

Not applicable.

## Abbreviations

ATP1A4	Na^+^-K^+^ ATPase α4 isoform
ADAM32	ADAM metallopeptidase domain 32
BSE	breeding soundness examinations
cAMP	cyclic adenosine monophosphate
cBiMPS	5,6-dichloro-1-β-D-ribofuranosylbenzimidazole-3′,5′-monophosphorothioate
[Ca^2+^]_i_	cytosolic/free intracellular calcium
DAG	diacylglycerol
EGFR	epidermal growth factor receptor
ERK	extracellular signal-regulated kinases
IP3	inositol 1,4,5-trisphosphate
ICSI	intracytoplasmic sperm injection
MAPK	microtubule-associated protein kinases
NCX	Na^+^/Ca^2+^ ion channel
NKA	Na^+^-K^+^ ATPase
NHE	Na^+^/H^+^ exchanger
PIP_2_	phosphatidylinositol 4,5-bisphosphate
PKA	protein kinase A
PLC/PKC	phospholipase C/protein kinase C
PI3K	phosphoinositide 3-kinase
PDK	3-phosphoinositide-dependent kinase
